# Association between Plasminogen Activator Inhibitor-1 -675 4G/5G Polymorphism and Sepsis: A Meta-Analysis

**DOI:** 10.1371/journal.pone.0054883

**Published:** 2013-01-30

**Authors:** Li Li, Wei Nie, Hongfeng Zhou, Weifeng Yuan, Weifeng Li, Wenjie Huang

**Affiliations:** 1 Department of Respiratory Medicine, Guangzhou General Hospital of Guangzhou Military Command, Guangzhou, Guangdong Province, China; 2 Department of Respiratory Disease, Shanghai Changzheng Hospital, Second Military Medical University, Shanghai, China; 3 Department of Anesthesiology, First Municipal People’s Hospital of Guangzhou, Guangzhou, Guangdong Province, China; University of Montreal, Canada

## Abstract

**Background:**

Several studies have evaluated the association between *plasminogen activator inhibitor-1* (*PAI-1*) -675 4G/5G polymorphism and sepsis in different populations. However, the available results are conflicting.

**Methods:**

A search of Pubmed and EMBASE databases was performed to identify relevant studies for inclusion in the meta-analysis. Odds ratios (ORs) and corresponding 95% confidence intervals (CIs) were determined using a random-effects model.

**Results:**

Twelve case-control studies and three cohort studies were included. Overall, a significant association between 4G/5G polymorphism and sepsis risk was observed for 4G/4G vs. 4G/5G +5G/5G (OR = 1.30, 95% CI 1.08–1.56, *P = *0.006). In addition, there was a significant association between *PAI-1* 4G/5G polymorphism and sepsis-related mortality (OR = 1.72, 95% CI 1.27–2.33, *P = *0.0005). In subgroup analyses, increased sepsis risk and mortality risk were found in Caucasians and in patients with sepsis.

**Conclusions:**

This meta-analysis suggested that the *PAI-1* -675 4G/5G polymorphism was a risk factor for sepsis and sepsis mortality.

## Introduction

Sepsis is a major public health problem that is responsible for an estimated economic burden of nearly 17 billion dollars annually in the United States. Despite the development of effective antibiotics and supportive care, sepsis remains the leading cause of death in critically ill patients [1]. Therefore, predictive markers to identify high-risk patients are urgently needed for early detection and preventive care. Recently, a number of investigators have begun the search for genetic risk factors that influence clinical outcomes in sepsis, and the *plasminogen activator inhibitor-1* (*PAI-1*) gene has been studied extensively.

PAI-1, a member of the serine protease inhibitor (serpin) family, is associated with the severity and outcome of sepsis [2]. Zeerleder and coworkers reported that PAI-1 levels were significantly higher in septic shock patients than in severe sepsis patients [3]. Furthermore, plasma PAI-1 was significantly higher in septic disseminated intravascular coagulation (DIC) patients than in nonseptic DIC patients, and its elevation was an independent risk factor for mortality in the septic DIC group [4]. In patients with meningococcal sepsis, previous studies showed that concentrations of PAI-1 were markedly elevated and there was a significant correlation between PAI-1 levels and mortality [5], [6]. Taken together, these results suggest that PAI-1 may play a pivotal role in the pathogenesis of sepsis.

The human *PAI-1* gene is located on chromosome 7, which contains a 4G/5G polymorphism located within the promoter region, 675 base pairs upstream of the transcription start site. This polymorphism has a role in the regulation of PAI-1 levels [7]. Recently, a number of research groups have studied this polymorphism as a potential susceptibility factor for sepsis. Several studies assessed the association between *PAI-1* -675 4G/5G polymorphism and the risk and outcomes of sepsis [6], [8]–[21]. However, the results were not consistent and remained inconclusive. As most studies had relatively small sample sizes, we performed a meta-analysis to determine whether *PAI-1* -675 4G/5G polymorphism was associated with an increased risk of sepsis or higher sepsis mortality. To our knowledge, this was the first meta-analysis of the association between *PAI-1* -675 4G/5G polymorphism and sepsis risk and mortality.

## Methods

### Publication Search

Relevant studies were identified by searching Pubmed and EMBASE databases up to September 2012. The following search terms were used: sepsis and (plasminogen activator inhibitor-1 or PAI-1 or SERPINE1) and (polymorphism or mutation or variant). All the searched studies were retrieved, and their references were also checked for other relevant publications. Review articles were also searched to find additional eligible studies. No publication date or language restrictions were applied.

### Inclusion and Exclusion Criteria

Studies fulfilling the following selection criteria were included in this meta-analysis: (1) they evaluated the association between -675 4G/5G polymorphism in *PAI-1* gene and risk of sepsis or sepsis mortality; (2) they were case-control or cohort studies; (3) they should report *PAI-1* genotype as 4G/4G vs. 4G/5G vs. 5G/5G. Studies were excluded if one of the following criteria existed: (1) the studies were not relevant to -675 4G/5G polymorphism, PAI-1, sepsis, or mortality; (2) they were non-clinical; (3) they were reviews or comments. For overlapping studies, only the one with the largest sample size was included.

### Qualitative Assessment

Two authors (Li and Nie) independently assessed the quality of each study. Any disagreement was resolved by consensus. Quality assessment scores of genetic association studies of human sepsis were used to assess the quality of the selected articles [22]. This quality scoring system was based on both traditional epidemiologic considerations and genetic issues. Total scores ranged from 0 (worst) to 9 (best) for cohort studies and 0 (worst) to 10 (best) for case-control studies.

### Data Extraction

The following variables were extracted from each study if available: first author’s surname, publication year, ethnicity of study participants, study design, age, type of sepsis, numbers of cases and controls, and genotype numbers in cases and controls. Information was carefully entered into predesigned data collection forms, independently by two of the investigators (Li and Nie). The accuracy of the data was verified by comparing collection forms from each investigator. Any discrepancy was resolved by discussion, or a third author (Huang) would assess these articles.

### Statistical Analysis

Where the data from at least three similar studies were available, a meta-analysis was performed. OR and 95% CI were employed to evaluate the strength of the association between -675 4G/5G polymorphism and the risk of sepsis and death. ORs were calculated for the genotypes: 4G/4G vs. 5G/5G (OR1), 4G/5G vs. 5G/5G (OR2), and 4G/4G vs. 4G/5G (OR3) for the -675 4G/5G polymorphism. These pairwise differences were used to indicate the most appropriate genetic model as follows: if OR1 =  OR3 ≠ 1 and OR2 = 1, then a recessive model was suggested; if OR1 =  OR2 ≠ 1 and OR3 = 1, then a dominant model was suggested; if OR2 = 1/OR3 ≠ 1 and OR1 = 1, then a complete overdominant model was suggested; if OR1> OR2>1 and OR1> OR3>1 (or OR1< OR2<1 and OR1< OR3<1), then a codominant model was suggested [23]–[25]. Once the best genetic model was identified, this model was used to collapse the three genotypes into two groups (except in the case of a codominant model) and to pool the results again. A random-effects model, using the Mantel-Haenszel method, was used to calculate the pooled ORs. The statistical significance of OR was determined with the *Z* test.

Departure from Hardy-Weinberg equilibrium (HWE) in controls was tested by the Chi-square test. The Q statistic and the *I* ^2^ statistic were used to assess the degree of heterogeneity among the studies included in the meta-analysis. Subgroup analyses were carried out by ethnicity and type of sepsis. Sensitivity analysis was performed through sequentially excluded individual studies to assess the stability of the results. The potential publication bias was examined visually in a funnel plot of log [OR] against its standard error (SE), and the degree of asymmetry was tested using Egger’s test [26].

All statistical tests were performed using Revman 5.1 software (Nordic Cochrane Center, Copenhagen, Denmark) and STATA 11.0 software (Stata Corporation, College Station, TX, USA). A *P* value <0.05 was considered statistically significant, except for tests of heterogeneity where a level of 0.10 was used.

## Results

### Study Characteristics

**Figure 1:**
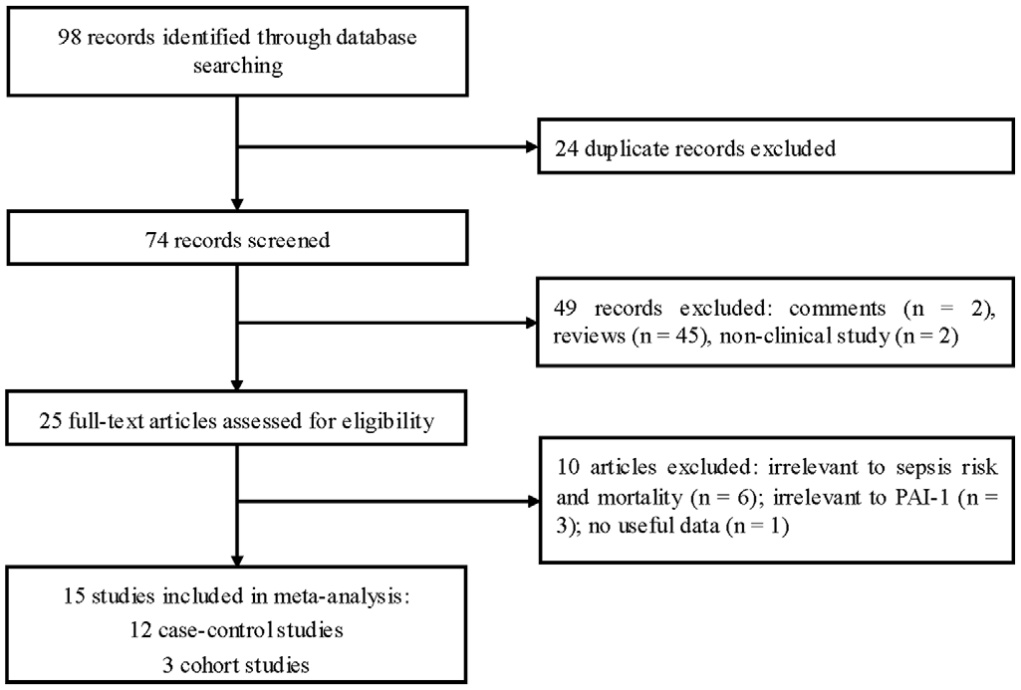
Flow of study identification, inclusion, and exclusion.

The literature search identified a total of 98 records. After removing 24 duplications and reading the abstracts, a further 49 articles were excluded. After reading the full text of the remaining articles, 10 were then excluded and 15 articles remained. The study selection process is shown in **Figure 1**. Finally, 12 case-control studies and 3 cohort studies were included in our meta-analysis [6], [8]–[21]. Thirteen studies were conducted in Caucasian populations, and two in Asian populations. Most of the studies comprised adult patients, but five studies focused on pediatric patients. Sepsis was defined as sepsis (eight studies), severe sepsis (two studies), septic shock (two studies), and mixed (two studies). The quality scores of most studies ranged from 5 to 8, suggesting high quality. We did not assess the quality of the study conducted by Lorente et al. [19], because insufficient information could be extracted from that study. The characteristics of the selected studies are presented in **Table 1**. Genotype numbers and HWE examination results are shown in **Table 2**.

**Table 1:** Characteristics of the studies included in the meta-analysis.

		Study		Age	Sepsis	Case	Control	Quality
First author	Year	design	Ethnicity	group	type	number (n)	number (n)	score
Hermans [6]	1999	Case-control	Caucasian	Pediatric	Sepsis	154	226	6
Westendorp [8]	1999	Case-control	Caucasian	Adult	Septic shock	85	131	6
Menges [9]	2001	Case-control	Caucasian	Adult	Sepsis	29	32	6
Haralambous [10]	2003	Case-control	Caucasian	Pediatric	Sepsis	230	155	6
Geishofer [11]	2005	Case-control	Caucasian	Pediatric	Sepsis	137	316	5
Zhan [12]	2005	Case-control	Asian	Adult	Sepsis	89	100	5
Sipahi [13]	2006	Case-control	Caucasian	Pediatric	Severe sepsis	42	113	5
García-Segarra [14]	2007	Case-control	Caucasian	Adult	Mixed	165	80	7
Jessen [15]	2007	Cohort	Caucasian	Adult	Mixed	317	NA	5
Wei [16]	2008	Case-control	Asian	Pediatric	Sepsis	148	181	6
Henckaerts [17]	2009	Case-control	Caucasian	Adult	Sepsis	395	555	8
Wingeyer [18]	2009	Case-control	Caucasian	Adult	Sepsis	166	136	5
Lorente [19]	2009	Cohort	Caucasian	NA	Severe sepsis	122	NA	NA
Madach [20]	2010	Cohort	Caucasian	Adult	Mixed	207	NA	7
Wingeyer [21]	2012	Case-control	Caucasian	Adult	Sepsis	166	214	5

NA, not available.

**Table 2:** Distribution of *PAI-1* genotype among patients with sepsis and controls.

	Sepsis			Control			Hardy-Weinberg
First author	4G/4G	4G/5G	5G/5G	4G/4G	4G/5G	5G/5G	equilibrium
Hermans [6]	40	85	29	59	114	53	Yes
Westendorp [8]	31	45	9	35	69	27	Yes
Menges [9]	16	11	2	13	14	5	Yes
Haralambous [10]	78	108	44	48	74	33	Yes
Geishofer [11]	45	67	25	91	149	76	Yes
Zhan [12]	39	37	13	25	50	25	Yes
Sipahi [13]	23	14	5	28	57	28	Yes
García-Segarra [14]	37	85	43	22	42	16	Yes
Jessen [15]	90	183	44	NA	NA	NA	NA
Wei [16]	47	68	33	50	91	40	Yes
Henckaerts [17]	140	177	78	162	280	113	Yes
Wingeyer [18]	42	54	70	33	64	39	Yes
Lorente [19]	23	60	39	NA	NA	NA	NA
Madach [20]	63	105	39	NA	NA	NA	NA
Wingeyer [21]	41	58	67	49	99	66	Yes

NA, not available.

### Quantitative Data Synthesis

**Figure 2:**
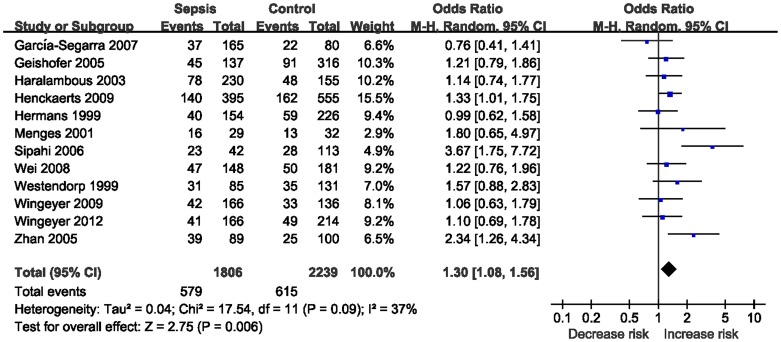
Meta-analysis for the association between sepsis risk and the *PAI-1* 4G/5G polymorphism (4G/4G vs. 4G/5G +5G/5G).

Twelve studies determined the association between -675 4G/5G polymorphism and sepsis risk [6], [8]–[14], [16]–[18], [21]. Total sample sizes for sepsis and control groups were 1806 and 2239, respectively. The estimated OR1, OR2 and OR3 were 1.30, 0.97, and 1.30, respectively (**Table 3**). These estimates suggested a recessive genetic model, and therefore 4G/4G was compared with 4G/5G and 5G/5G. The pooled OR in this analysis was 1.30 (95% CI 1.08–1.56; *P* = 0.006) (**Figure 2**). This result suggested that the 4G/4G genotype was significantly associated with sepsis risk. In the subgroup analysis by ethnicity, significant associations were found among Caucasians (OR = 1.24; 95% CI 1.02–1.51; *P* = 0.03). Subgroup analysis was also performed by sepsis type. Increased risk was found among the patients with sepsis (OR = 1.25; 95% CI 1.07–1.45; *P* = 0.004).

**Table 3:** Summary of meta-analysis results.

		No. of	Test of association				Heterogeneity		
Comparison	Study	studies	OR (95% CI)	*Z*	*P* Value	Model	*χ* ^2^	*P* Value	*I* ^2^ (%)
4G/4G vs. 5G/5G (OR1)	Overall	12	1.30	1.88	0.06	R	22.52	0.02	51.0
			(0.99–1.72)						
4G/5G vs. 5G/5G (OR2)	Overall	12	0.97	0.22	0.83	R	20.15	0.04	45.0
			(0.77–1.24)						
4G/4G vs. 4G/5G (OR3)	Overall	12	1.30	3.15	0.002	R	12.85	0.30	14.0
			(1.11–1.54)						
Risk of sepsis									
4G/4G vs. 4G/5G +5G/5G	Overall	12	1.30	2.75	0.006	R	17.54	0.09	37.0
			(1.08–1.56)						
	Caucasian	10	1.24	2.20	0.03	R	13.65	0.14	34.0
			(1.02–1.51)						
	Sepsis	9	1.25	2.88	0.004	R	6.44	0.60	0.0
			(1.07–1.45)						
Risk of mortality									
4G/4G vs. 4G/5G +5G/5G	Overall	12	1.72	3.48	0.0005	R	20.89	0.03	47.0
			(1.27–2.33)						
	Caucasian	10	1.59	2.88	0.004	R	16.68	0.05	46.0
			(1.16–2.17)						
	Sepsis	7	2.06	3.57	0.0004	R	12.10	0.06	50.0
			(1.38–3.05)						

vs., versus; R, random-effects model.

**Figure 3:**
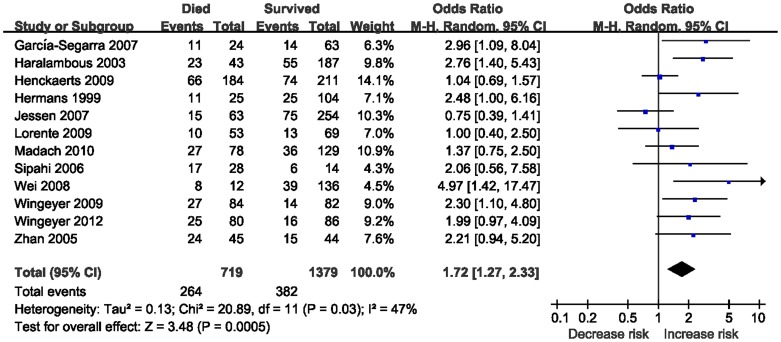
Meta-analysis for the association between mortality risk and the *PAI-1* 4G/5G polymorphism (4G/4G vs. 4G/5G +5G/5G).

Twelve studies identified an association between -675 4G/5G polymorphism and sepsis-related mortality risk [6], [10], [12]–[21]. The total sample included 2098 patients. **Figure 3** shows a significant association between -675 4G/5G polymorphism and sepsis-related mortality with an OR of 1.72 (95% CI 1.27–2.33; *P* = 0.0005). In the subgroup analysis by ethnicity, significant association was also found among Caucasians with the 4G/4G genotype (OR = 1.59; 95% CI 1.16–2.17; *P* = 0.004). Stratification by sepsis type showed that patients with sepsis carrying 4G/4G genotype were associated with an increased mortality risk (OR = 2.06; 95% CI 1.38–3.05; *P* = 0.0004). Summary results of comparisons are listed in **Table 3**.

### Heterogeneity Analysis

**Figure 4:**
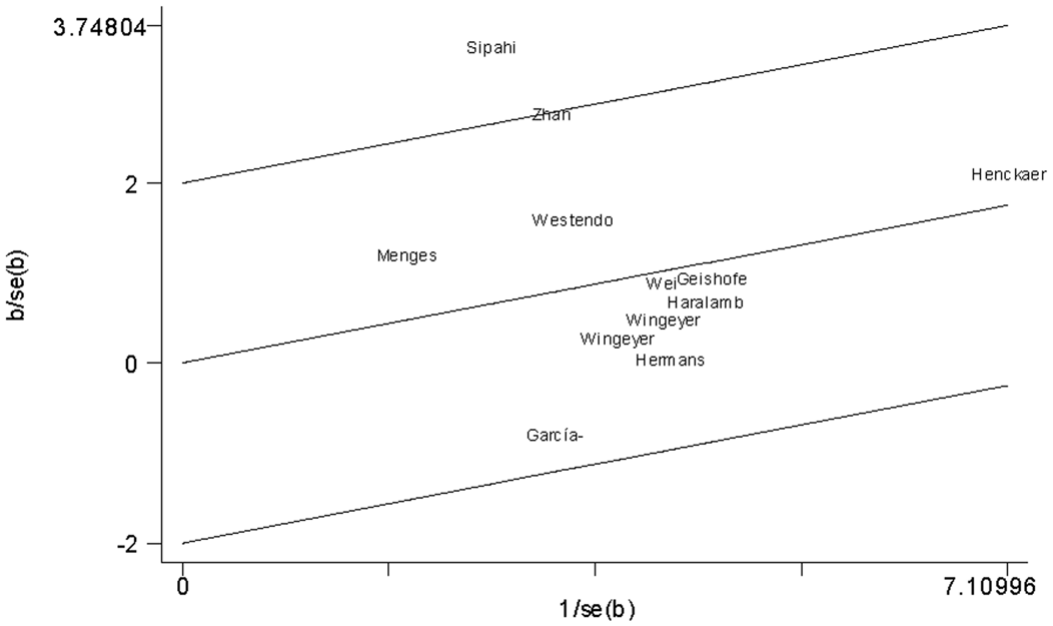
Galbraith plot of *PAI-1* 4G/5G polymorphism and sepsis risk.

For sepsis risk, there was a statistically significant between-study heterogeneity in the recessive genetic model (*I^2^* = 37%). Galbraith plots were used to explain the heterogeneity. The study performed by Sipahi et al. [13] was the outlier in the recessive genetic model (**Figure 4**). After excluding this study, the between-study heterogeneity effectively decreased and there was no obvious heterogeneity among the 11 remaining studies (*I^2^* = 0%).

**Figure 5:**
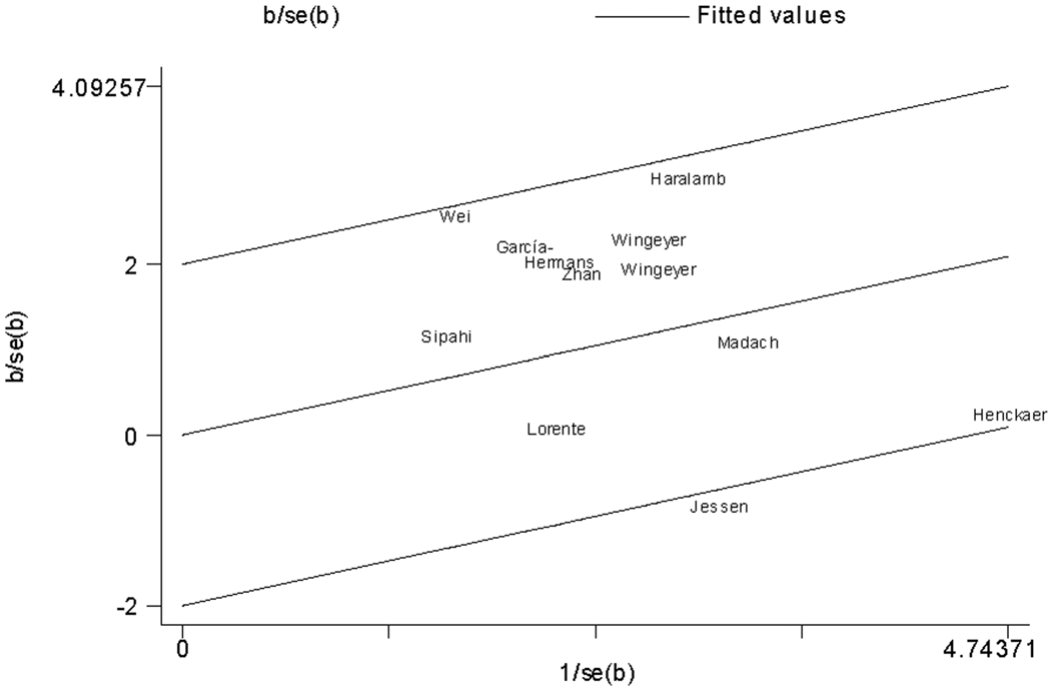
Galbraith plot of *PAI-1* 4G/5G polymorphism and sepsis-related mortality risk.

For mortality risk, there was also statistically significant between-study heterogeneity (*I^2^* = 47%). Galbraith plots found one study [15] as the outlier and the possible major source of heterogeneity (**Figure 5**). When the study was excluded, the *I^2^* value was 34%.

### Sensitivity Analysis

To evaluate the stability of the results of the meta-analysis, sensitivity analyses were performed through sequentially omitted individual studies. None of the results were materially changed, which suggested the robustness of our results (data not shown).

### Cumulative Meta-analysis

**Figure 6:**
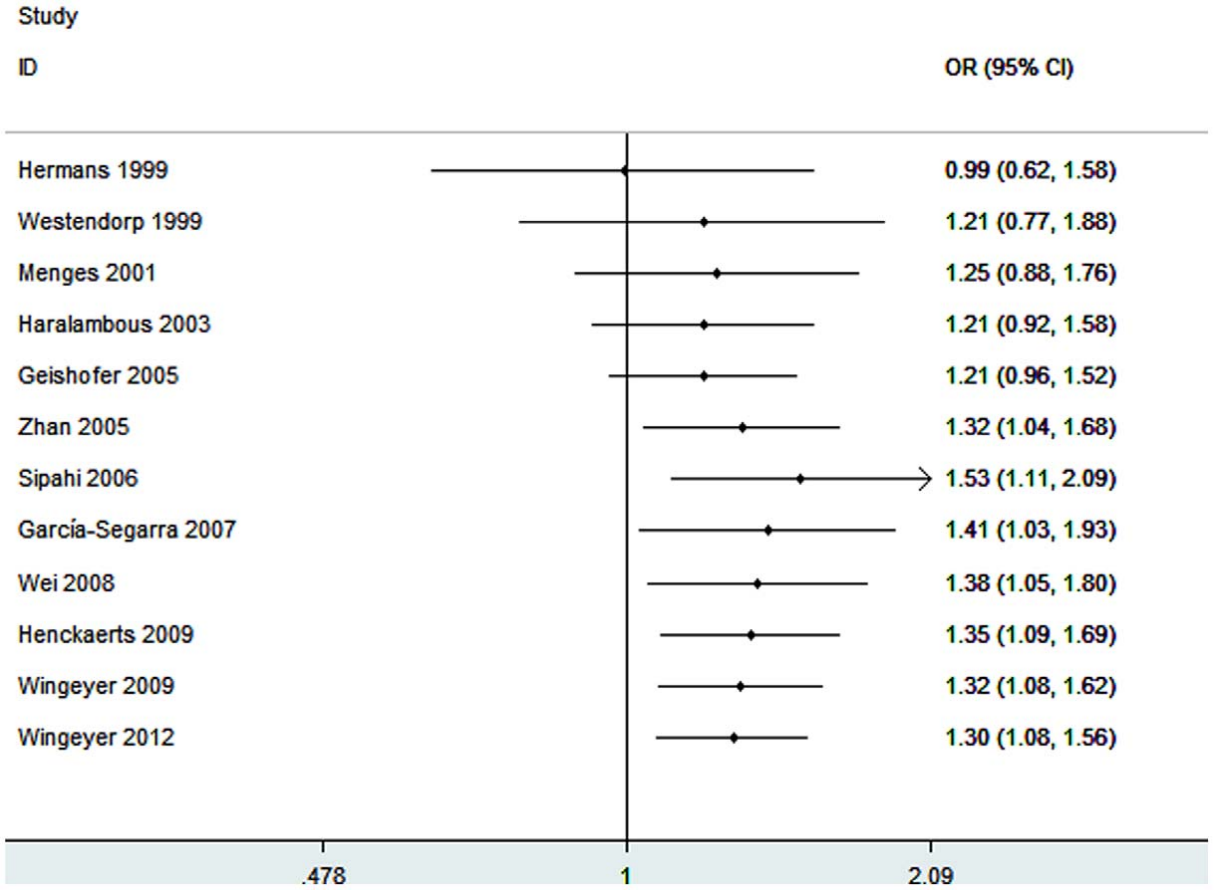
Cumulative meta-analysis of associations between the *PAI-1* 4G/5G polymorphism and sepsis risk.

**Figure 7:**
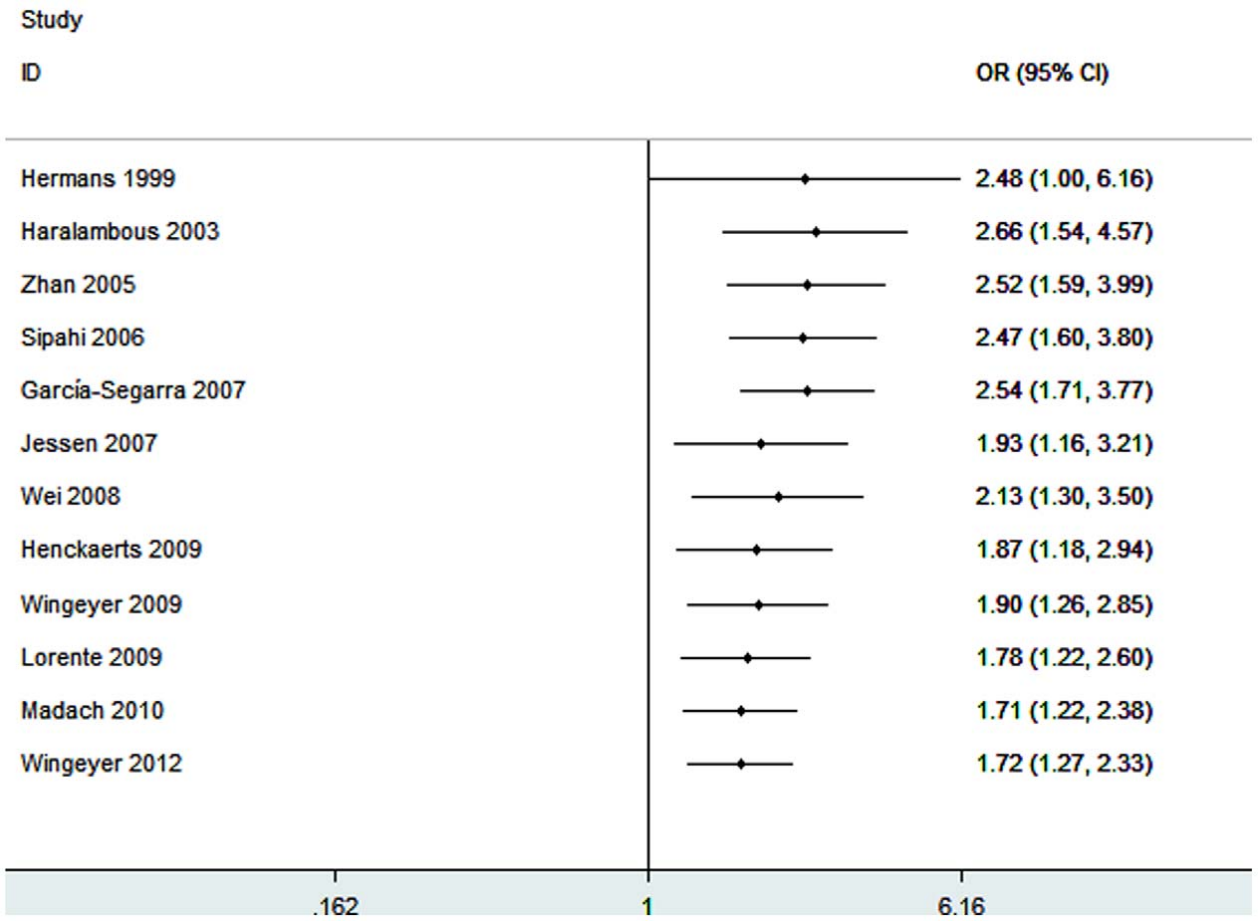
Cumulative meta-analysis of associations between the *PAI-1* 4G/5G polymorphism and sepsis-related mortality risk.

Cumulative meta-analyses of the two associations were performed via the assortment of studies by publication time. As shown in **Figure 6** and **Figure 7**, inclinations toward significant associations were evident with each addition of more data over time. The results showed that the pooled ORs tended to be stable.

### Publication Bias

**Figure 8:**
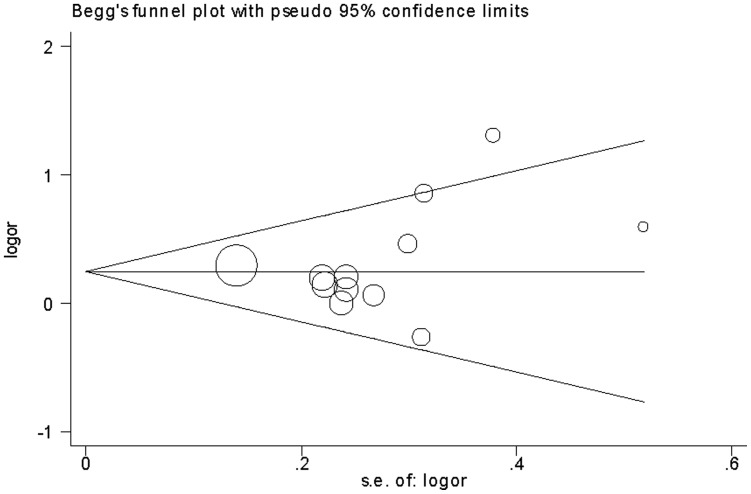
Begg's funnel plot for sepsis risk and the *PAI-1* 4G/5G polymorphism (4G/4G vs. 4G/5G +5G/5G).

**Figure 9:**
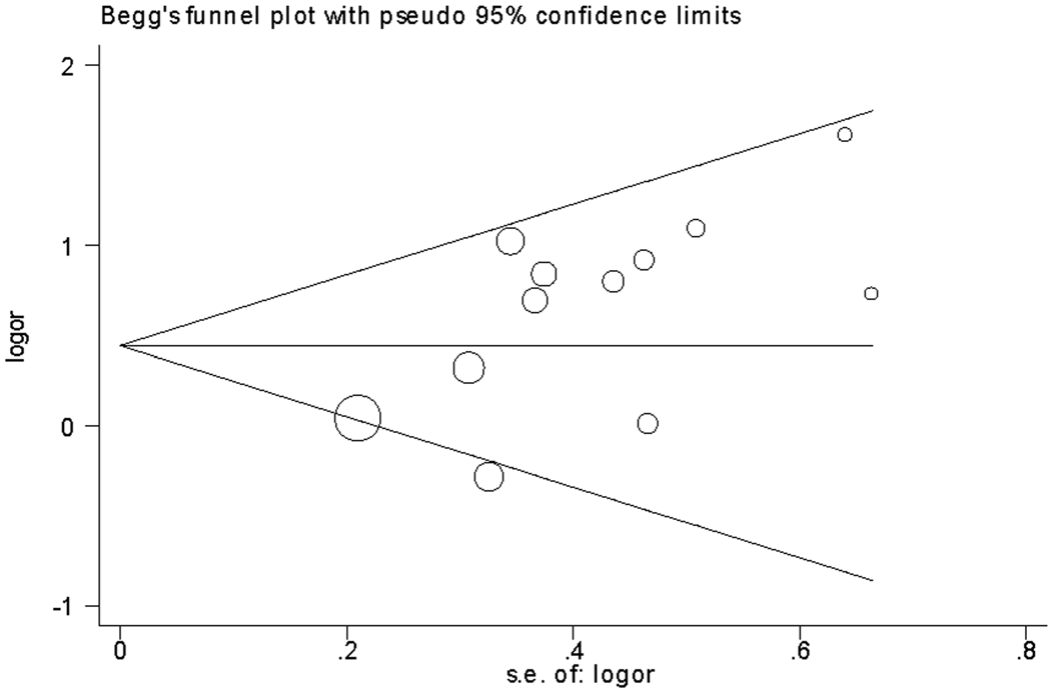
Begg's funnel plot for mortality risk and the *PAI-1* 4G/5G polymorphism (4G/4G vs. 4G/5G +5G/5G).

Publication bias was examined by funnel plots qualitatively and estimated by Egger’s test quantitatively. The shapes of the funnel plots seemed slightly asymmetrical (**Figure 8** and **Figure 9**). Egger’s test did not show evidence of publication bias for sepsis risk (*P* = 0.322). However, publication bias was found for mortality risk (*P* = 0.024).

## Discussion

Sepsis is a complex clinical syndrome that results from a systemic inflammatory response to bacteria and/or bacterial products [2]. Coagulation and inflammation are reciprocally interdependent and closely related in sepsis [27]. Haemostatic changes, ranging from mild laboratory alterations to DIC, are often observed during sepsis [28]. DIC is an independent predictor of poor outcome in patients with severe sepsis [29], [30]. The imbalance between fibrin formation and deposition contributes to DIC [31]. PAI-1 is a principal inhibitor of tissue plasminogen activator (t-PA) and can be viewed as a potent inhibitor of fibrinolysis. Recently, Semeraro et al. [32] suggested that suppression of fibrinolysis due to PAI-1 was an important mechanism in DIC development. In addition, a growing number of studies confirmed that high levels of PAI-1 correlated closely with the severity of sepsis, and were predictive of adverse outcomes [33], [34]. Consequently, PAI-1 may play a critical role in the development of sepsis, and increased levels of PAI-1 may predict a high mortality risk. An early study reported that a functional mutation in the *PAI-1* gene (the 4G/5G polymorphism) could influence the expression of the *PAI-1* gene [35]. The 4G/4G genotype has been linked to higher PAI-1 level, compared with the 5G/5G genotype, with the heterozygous genotype associated with intermediate levels [36]. Therefore, we hypothesized that *PAI-1* -675 4G/5G polymorphism could influence the susceptibility to sepsis and sepsis-related mortality.

In our meta-analysis, we found that the 4G/4G genotype was a modest risk factor for developing sepsis in the overall study population. The results revealed that carriers of the 4G/4G genotype had a 30% increased sepsis risk compared with individuals carrying the 5G allele (4G/5G +5G/5G). In the subgroup analysis, we noted that Caucasians carrying the 4G/4G genotype had an increased sepsis risk. There were only two studies on Asians for this polymorphism [12], [16]. Therefore, subgroup analysis was not performed in the Asians subgroup. More studies in Asian populations are needed to evaluate the effect of -675 4G/5G polymorphism on sepsis risk. In addition, we carried out subgroup analysis by sepsis type. We found that patients in sepsis subgroup who carrying 4G/4G genotype had an increased disease risk. Since there were only three studies performed in patients with severe sepsis or septic shock, subgroup analyses could not be conducted and more studies should be designed to analyze these conditions. A significant association was found between *PAI-1* -675 4G/5G polymorphism and sepsis-related mortality. We found that septic patients with the 4G/4G genotype had a 72% increased mortality risk compared to patients with 4G/5G genotype or 5G/5G genotype. Similarly, significant results were also noted in the Caucasian subgroup and sepsis subgroup. Since our meta-analysis included no more than two Asian studies, severe sepsis, or septic shock populations, any positive association between these conditions and sepsis-related mortality could not be ruled out, because a small sample size may have insufficient statistical power to detect a slight effect. These associations require further study.

There were modest heterogeneities in the overall comparisons for *PAI-1* -675 4G/5G polymorphism. Galbraith plots were used to explore the sources of heterogeneity. We found that all *I^2^* values were decreased after excluding the outliers. The results suggested that the two outlying studies [13], [15] might be the major source of the heterogeneity. However, heterogeneity did not seem to influence the results, because the significance of the result was not altered after excluding the outliers. Moreover, we carried out sensitivity analyses. Removal of each study did not alter the associations with sepsis risk and mortality risk, suggesting the reliability of these results. The cumulative meta-analyses showed a trend of more marked associations between *PAI-1* -675 4G/5G polymorphism and increased risk of sepsis and mortality as data accumulated each year. This procedure also proved that our results were robust.

Salanti et al. [37] suggested that false-negative results may be suppressed or false-positive results magnified. Thus, the results of meta-analyses might be influenced by publication bias. Although Egger’s test did not show significant publication bias for sepsis risk, we found the shape of the funnel plot was slightly asymmetrical. In addition, significant publication bias was observed for mortality risk. Thus, the results should be interpreted cautiously and more studies are still needed to confirm the findings from this meta-analysis.

Some limitations of this meta-analysis should be pointed out. First, the number of included studies in our meta-analysis was moderate. Second, most of the studies were conducted in Caucasian populations. Therefore, our results may be applicable only to this ethnic group. Third, sepsis is a complex disease, and many genes are associated with it [38], [39]. However, we could not address gene-gene interactions in this meta-analysis due to the lack of the related information. Fourth, the overall outcome was based on unadjusted data, whereas a more precise analysis could be performed if individual data were available to allow adjustment. Finally, because only the studies that were indexed by the selected databases were included in our meta-analysis, some relevant published studies may not have been included, which may have biased our results.

In conclusion, this meta-analysis suggested that *PAI-1* -675 4G/5G polymorphism may represent a risk factor for sepsis and sepsis-related mortality. Well-designed studies with large sample sizes are needed to further evaluate the associations between this polymorphism and clinical outcomes of sepsis in various ethnic populations. Moreover, gene-gene interactions should also be considered in future studies.
